# Transcriptional control of Arabidopsis seed development

**DOI:** 10.1007/s00425-022-03870-x

**Published:** 2022-03-23

**Authors:** Subodh Verma, Venkata Pardha Saradhi Attuluri, Hélène S. Robert

**Affiliations:** Mendel Centre for Genomics and Proteomics of Plants Systems, CEITEC MU - Central European Institute of Technology, Masaryk University, Brno, Czech Republic

**Keywords:** Arabidopsis, Seed, Embryo patterning, Transcription factors, Maturation

## Abstract

**Main conclusion:**

The entire process of embryo development is under the tight control of various transcription factors. Together with other proteins, they act in a combinatorial manner and control distinct events during embryo development.

**Abstract:**

Seed development is a complex process that proceeds through sequences of events regulated by the interplay of various genes, prominent among them being the transcription factors (TFs). The members of WOX, HD-ZIP III, ARF, and CUC families have a preferential role in embryonic patterning. While WOX TFs are required for initiating body axis, HD-ZIP III TFs and CUCs establish bilateral symmetry and SAM. And ARF5 performs a major role during embryonic root, ground tissue, and vasculature development. TFs such as LEC1, ABI3, FUS3, and LEC2 (LAFL) are considered the master regulators of seed maturation. Furthermore, several new TFs involved in seed storage reserves and dormancy have been identified in the last few years. Their association with those master regulators has been established in the model plant Arabidopsis. Also, using chromatin immunoprecipitation (ChIP) assay coupled with transcriptomics, genome-wide target genes of these master regulators have recently been proposed. Many seed-specific genes, including those encoding oleosins and albumins, have appeared as the direct target of LAFL. Also, several other TFs act downstream of LAFL TFs and perform their function during maturation. In this review, the function of different TFs in different phases of early embryogenesis and maturation is discussed in detail, including information about their genetic and molecular interactors and target genes. Such knowledge can further be leveraged to understand and manipulate the regulatory mechanisms involved in seed development. In addition, the genomics approaches and their utilization to identify TFs aiming to study embryo development are discussed.

## Introduction

**Fig. 1:**
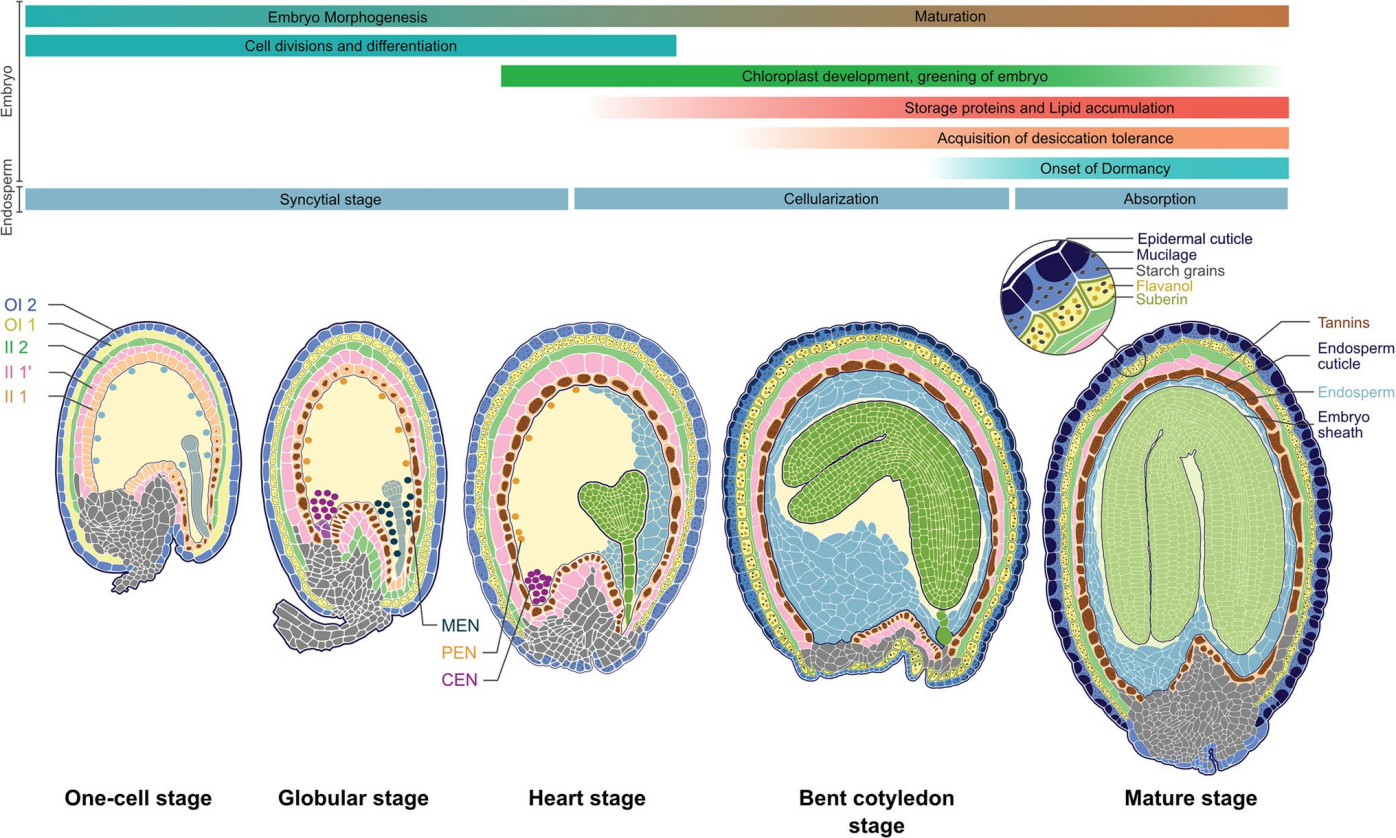
Overall depiction of Arabidopsis seed development. The development of three components of the seed (seed coat, endosperm, and embryo) is illustrated. All five layers of the seed coat derived from ovules integuments are shown in different colors. Polymers accumulated in the seed coat layers are shown. Endosperm development is defined by the syncytial stage, followed by cellularization and absorption. Differentiation of the three mitotic domains of the endosperm (micropylar, peripheral, and chalazal) is depicted with nuclei in different colors in the globular stage. Embryo development stages are shown from one-cell to mature stages. At the top of the figure, bars represent the major events that occur during embryo and endosperm development. MEN, micropylar endosperm nuclei; PEN, peripheral endosperm nuclei; CEN, chalazal endosperm nuclei

In most angiosperms, the seed is an outcome of a double fertilization process, in which one of the two sperm nuclei fuses with the egg cell to produce a diploid zygote, and the second sperm nucleus fuses with the binucleate central cell to generate the triploid endosperm (Baroux and Grossniklaus 2019). Subsequently, the single cellular zygote undergoes highly coordinated cell divisions and cellular differentiation to develop into a multicellular embryo in a process termed embryogenesis (Verma et al. 2021). In many dicots, including Arabidopsis, the endosperm develops as a coenocyte (series of mitosis without cytokinesis) followed by endosperm cellularization (Fig. 1; Brown et al. 1999; Li and Berger 2012; Batista et al. 2019). Later, in Arabidopsis, the endosperm is absorbed in part by the growing embryo. The seed coat is developed from the ovule’s integuments and consists of five cell layers. Two cell layers are derived from the outer integuments (OI): OI2 and OI1, and three cell layers are derived from the inner integuments (II): II2, II1′ and II1 (Fig. 1). During the development of the seed, cells of the outermost layer, i.e., OI2, produce mucilage which accumulates specifically at the outer corners of the cell. Besides, both outer integument cell layers (OI1 and OI2) produce starch granules, flavanols, and suberin (a lipophilic polymer) (Golz et al. 2018; Francoz et al. 2018). In contrast, cells of the innermost layer accumulate proanthocyanidins (PAs) which later oxidize and give the characteristic brown colour to the seed coat. In the end, the mature seed contains a filial embryo, a single-cell endosperm layer, and a protecting covering, i.e., a seed coat developed from the ovule's integuments.

Overall, embryo development can be classified into two distinct phases, (i) morphogenesis and (ii) maturation (Fig. 1; Goldberg et al. 1994; O'Neill et al. 2019; Jo et al. 2019). The morphogenesis phase begins immediately after fertilization and lasts until the late-heart stage. This phase is characterized by highly coordinated cell divisions and differentiation, during which the basic body organization of the embryo is established. By contrast, cell division and proliferation are ceased during embryo maturation. However, cell expansion occurs along with the accumulation of seed storage reserves such as carbohydrates, proteins, and fatty acids (Jo et al. 2019). Embryo maturation partially overlaps with the morphogenesis phase. It begins around the early-heart stage and lasts until the seed is filled with nutrients, becomes dry, and acquires a dormant state (e.g., seed maturation). It has been observed that the developmental stage, not the time elapsed since fertilization, determines the onset of maturation (O'Neill et al. 2019).

Seed development depends on the spatiotemporal expression of various genes involved in different processes occurring during this period, including cell division, differentiation, seed filling, desiccation, and dormancy (Le et al. 2010). Therefore, control of the spatiotemporal pattern and expression levels of such genes is crucial, which results from the regulation of their transcription by different transcription factors (TFs). TFs are critical regulatory proteins that act in a combinatorial manner together with other proteins to orchestrate this transcriptional regulation (Agarwal et al. 2011). They work through binding to specific DNA sequences (*cis*-regulatory elements) present over the promoters of their target genes (Spitz and Furlong 2012). These regulatory proteins are themselves regulated by other TFs, miRNAs, hormones, etc., thereby creating multiple layers and networks of regulation to control a particular developmental aspect. Over the last few years, several TFs regulating seed and/or embryo development have been identified using genetics and genomics approaches in different plant species (Wu et al. 2020; Ren et al. 2021; Hofmann et al. 2019). However, the mechanisms through which they perform their function, including their interacting partners, target promoters, etc., are still not clear for most of them.

This review aims to enhance our understanding of transcriptional regulation of the distinct events of embryo morphogenesis and maturation by different classes of TFs in Arabidopsis seed development. Additionally, the identification of novel TFs using genome-wide approaches will be discussed, and how this will improve future research in studying the transcriptional landscape of seed development.

## Transcriptional control of early embryogenesis (morphogenesis)

Embryogenesis begins when the zygote undergoes an asymmetric division that generates a small apical cell and a large basal cell (Capron et al. 2009). The apical cell gives rise to the spherical embryo proper (proembryo) that will create most of the mature embryo. Two rounds of longitudinal and one round of transverse divisions convert the apical cell into an eight-celled embryo. In this stage, two domains, termed the upper tier and lower tier, are distinguished. The basal cell divides transversely and gives rise to a 7–9 celled filamentous suspensor. Later, the uppermost suspensor cell is specified as hypophysis, which ultimately protrudes into the embryo. The upper tier gives rise to the cotyledons and shoot apical meristem (SAM). The lower tier and hypophysis generate the cotyledons' abaxial part, hypocotyl, root apical meristem (RAM), and embryonic root. After a periclinal division, a 16-celled embryo results from the first visible cell differentiation when the outermost layer is specified as protoderm. Subsequent divisions give the embryo a globular appearance. At this stage, precursor cells of ground tissue and vascular tissue are specified. Later, with the development of cotyledon primordia, the embryo takes the shape of a heart. At this stage, SAM is established, cotyledons are formed, and they begin to elongate, marking the beginning of the maturation phase.

### Transcription factors for initiating body axis

**Fig. 2:**
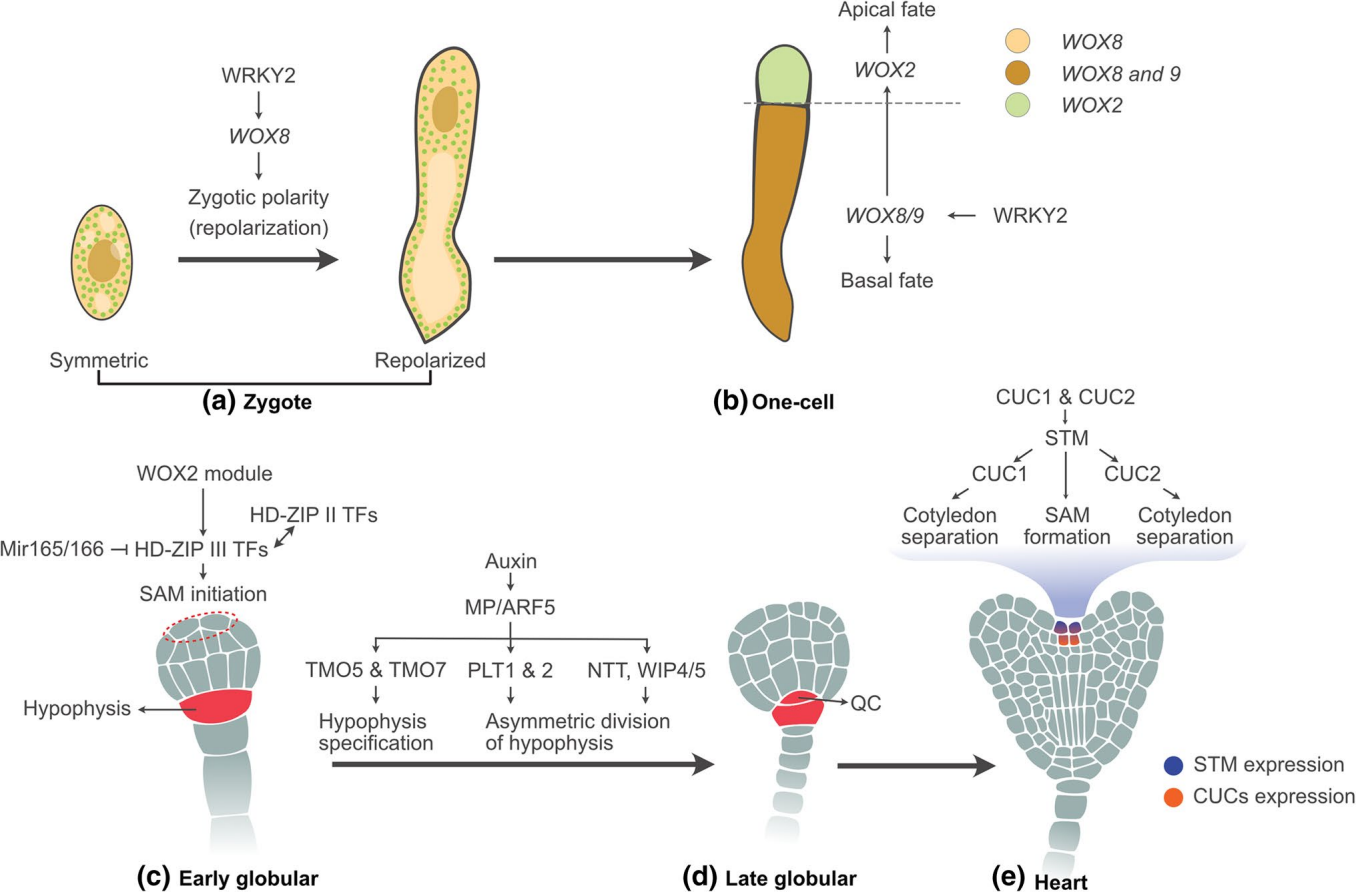
Transcription factors involved in embryo patterning. **A** In the zygote, WRKY2 activates the expression of *WOX8* to promote the polarization of the nucleus (brown) and vacuoles (light yellow) and asymmetric division of the zygote. **B** In the resulting asymmetric embryo, WRKY2-WOX8/9 non-cell-autonomously regulates the development of the embryo proper by activating *WOX2* expression (green) in the apical region. **C** At the early globular stage, the WOX2 module (WOX1/2/3/5) is required for the initiation of SAM. The WOX2 module activates the expression of HD-ZIP III TFs to protect the shoot meristem stem cells from differentiation. The HD-ZIP III TFs are targeted by miRNA165/166. These TFs act with HD-ZIP II TFs to regulate apical patterning during embryogenesis. The uppermost suspensor cell is specified as hypophysis via auxin-dependent activation of TMO5 and TMO7 by MP/ARF5. **D** Other targets of ARF5 such as NTT, WIP4/5, and PLTs are required for asymmetric division of the hypophysis and correct specification of the QC at the late globular stage. **E** CUC TFs regulate the boundary formation between the developing cotyledons and act synergistically with STM to control SAM formation

The first asymmetric division of the zygote lays the foundation for apical-basal patterning in the embryo. Members of the WUSCHEL-RELATED HOMEOBOX (WOX) TFs play key roles during embryo patterning. Different WOX genes exhibit distinct expression patterns throughout embryogenesis. For instance, WOX8/STIMPY is expressed in the zygote, where another TF, i.e., WRKY2, regulates its expression. This WRKY2-WOX8 interaction is required for the polar distribution of the cell organelles in the zygote, creating the zygotic polarity required to make the first division asymmetric (Fig. 2A; Ueda et al. 2011). WOX2, co-expressed with WOX8 in the zygote, expresses in the apical cell after the asymmetric zygotic division. WOX2 is required to regulate the apical patterning and development of SAM during embryogenesis. In contrast, WOX8 is expressed in the basal cell along with its closest homolog WOX9/STIMPY-LIKE after the zygotic division, redundantly regulating the basal cell lineage (Fig. 2B). They also regulate the embryo proper by activating WOX2 expression in the apical region (Breuninger et al. 2008). A shift of WOX9 expression from hypophysis to the embryo proper's basal region indicates its function in establishing the lower tier domain identity (Haecker et al. 2004). Additionally, other WOX TF family members, such as WOX1, WOX3, and WOX5, expressed in cotyledon primordia, vascular primordia, and hypophysis, respectively, contribute to embryo patterning (Haecker et al. 2004; Breuninger et al. 2008).

### Transcription factors in regulating apical patterning

Members of the class III homeodomain-leucine zipper (HD-ZIP III) TF family are known regulators of apical patterning during embryogenesis (Fig. 2C; Emery et al. 2003; Prigge et al. 2005; Smith and Long 2010). This family consists of five genes that encode *PHABULOSA* (*PHB*), *PHAVOLUTA* (*PHV*), *REVOLUTA* (*REV*), *ARABIDOPSIS THALIANA HOMEOBOX-8 (ATHB8),* and *INCURVATA4/CORONA/CNA/ATHB15*. All five have been predicted as the targets of miRNA165/166 (Smith and Long 2010; Floyd and Bowman 2004; Mallory et al. 2004). They perform an overlapping function in establishing bilateral symmetry and shoot apical meristem, as demonstrated by the phenotypes of their mutant combinations (Emery et al. 2003; Prigge et al. 2005). For instance, *rev phb* double mutant embryos occasionally display patterning defects such as single or radially symmetric cotyledon. Such defects are more severe when combined with *phv* and/or *cna*. However, *athb8* mutation does not affect these patterning defects (Prigge et al. 2005). Besides, members of HD-ZIP II TFs, i.e., HOMEOBOX ARABIDOPSIS THALIANA 3 (HAT3), ARABIDOPSIS THALIANA HOMEOBOX 2 (ATHB2), and ATHB4, respond to changes in light conditions and regulate cotyledon development, thus the establishment of bilateral symmetry during embryogenesis (Turchi et al. 2013). In contrast to single mutants where defects are not obvious, *hat3 athb4* double mutants display aberrantly developed cotyledons such as fused or single, and/or vasculature-less cotyledons. These defects are attenuated by a gain-of-function mutation in *ATHB2*, suggesting that ATHB2 is redundant to HAT3 and ATHB4. Moreover, most *hat3 athb2 athb4* mutant seedlings lack an active SAM. The defects in cotyledon development and SAM activity are enhanced when *hat3 athb4* is combined with mutations in HD-ZIP III genes. This indicates that HD-ZIP II and HD-ZIP III proteins act in the same genetic pathway to establish bilateral symmetry in the embryo and control SAM activity in seedlings. The direct interaction of REV on the *ATHB2* promoter indicates that HD-ZIP III proteins control HD-ZIP II proteins' expression. These findings suggest that HD-ZIP II and HD-ZIP III TFs act in a combinatorial manner to regulate apical patterning during embryogenesis (Turchi et al. 2013).

Besides, PHB, PHV, and REV are thought to be involved in defining the adaxial fate of the embryo. Their expression in the adaxial regions of the cotyledons and vasculature in heart-stage embryos supports this notion (Emery et al. 2003; McConnell et al. 2001). Moreover, *phb phv rev* triple mutant embryos appear to be fully abaxialized (Emery et al. 2003). In addition, *KANADI (KAN)* genes that encode the members of the GARP TFs family are found to be involved in embryo patterning along the abaxial side of the cotyledons and hypocotyl (Eshed et al. 2004; Kerstetter et al. 2001; Izhaki and Bowman 2007). Their mutant combination (*kan1 kan2 kan4*) displays abnormal phenotypes in the region that will generate the abaxial side of the respective tissues. Further genetic analysis indicates that HD-ZIP III and KAN act antagonistically to establish abaxial-adaxial identity in the embryo (Izhaki and Bowman 2007; Emery et al. 2003). Furthermore, the YABBY (YAB) gene family members (FILAMENTOUS FLOWER (FIL), YAB2, YAB3) are expressed in the abaxial domain of cotyledons at the mid-heart stage indicating its role in specifying abaxial cell fate (Siegfried et al. 1999). Despite all this, a detailed mechanism of adaxial-abaxial polarity during embryogenesis is still lacking and needs to be addressed. AP2/ERF-type TFs, DORNRÖSCHEN (DRN), and its paralogues DORNRÖSCHEN-LIKE (DRNL) interact with HD-ZIP III TFs. They act redundantly to regulate embryonic cell patterning and cotyledon development (Chandler et al. 2007; Cole et al. 2009). DRN and DRNL exhibit overlapping expression patterns till the early-heart stage (Chandler et al. 2007). DRN is expressed throughout the embryo proper from the 2 to 16-cell stage. Later, it becomes restricted to the apical domain at the site of cotyledon development (globular stage), then to the cotyledon tips at the heart stage (Chandler et al. 2007; Cole et al. 2009). The *drn* and *drn drnl* mutants are also defective in the hypophysis division, which gives rise to the embryonic root meristem. However, this function appears inconsistent with their expression, suggesting their non-cell-autonomous action in embryonic root formation (Cole et al. 2009).

The transition from radial to bilateral symmetry appears when the two distinct cotyledon primordia arise from the apical part of the globular embryo. Boundary formation between the developing cotyledons is essential for the establishment of SAM which is regulated by a gene regulatory network involving NAC (NAM/ATAF1/ATAF2/CUC) family TFs, i.e., CUP-SHAPED COTYLEDON1 (CUC1), CUC2, and CUC3 (Fig. 2D, E; Aida et al. 1999; Takada et al. 2001; Vroemen et al. 2003). CUC TFs act synergistically with another TF of class 1 KNOTTED1-LIKE HOMEOBOX (KNOX) TF family, SHOOT MERISTEMLESS (STM), to control SAM formation. Consistent with their function, CUC and STM genes are expressed in the central strip by the late globular or heart stage. Furthermore, the expression of the three CUC genes is differentially regulated by WOX2 and WOX8 TFs during the establishment of the cotyledon boundary (Lie et al. 2012). It has been shown that WOX2 collectively with its redundant paralogs (i.e., WOX1, WOX3, and WOX5) is essential for the initiation of shoot meristem stem cells (Fig. 2C; Zhang et al. 2017). This module protects stem cells from differentiation by activating the expression of HD-ZIP III TFs in the presumptive SAM region. In addition, the WOX2 module reduces auxin activity by downregulating PIN1 expression in that region. This reduced auxin activity contributes to the initiation of stem cells therein.

### Transcription factors in embryonic root formation

The establishment of RAM is initiated with the specification of the extraembryonic/uppermost suspensor cell as hypophysis (Fig. 2C, D). It divides asymmetrically and generates the quiescent centre (QC), which functions in maintaining stem cells (Capron et al. 2009). The *monopteros* (*mp*) mutants that lack the activity of the gene encoding the ARF5 TF, fail to initiate root meristem (Berleth and Jurgens 1993; Hamann et al. 1999, 2002). ARF5 is a downstream component of the auxin response pathway. It forms a complex with AUX/IAA12 (BODENLOS). In response to auxin, ARF5 releases from this complex and regulates the expression of its target genes by binding to the auxin response element (AuxRE) containing the core sequence TGTCTC. Two genes encoding bHLH TFs, i.e., TARGET OF MONOPTEROS5 (TMO5) and TMO7, have been identified as the direct targets of ARF5/MP that function in MP-mediated embryonic root formation (Schlereth et al. 2010). Other targets of ARF5/MP such as the gene encoding the zinc finger TF, NO TRANSMITTING TRACT (NTT), and its paralogs WIP domain protein 4 (WIP4) and WIP5 are required for root meristem initiation (Crawford et al. 2015). AP2 domain-containing TFs PLETHORA1 (PLT1) and PLT2 are redundantly required for the correct specification of QC, leading to the maintenance of the stem cells in the root meristem (Aida et al. 2004). Their basal embryonic expression requires ARF5 and ARF7/NPH4. Besides, the TFs that belong to the GRAS family, i.e., SHORT-ROOT (SHR) and SCARECROW (SCR), are required for QC identity and stem cell maintenance (Aida et al. 2004). Both *SCR* and *SHR* have different expression domains in the embryo. *SCR* is expressed in the hypophysis and ground tissue precursors at the globular stage. Later, after the asymmetric division of the hypophysis, *SCR* is expressed in the QC and nearby ground tissue (Helariutta et al. 2000; Wysocka-Diller et al. 2000). In comparison, *SHR* is expressed in the precursors of the vasculature (stele) but not in the hypophysis and QC (Helariutta et al. 2000). Nevertheless, in contrast to its expression region, SHR proteins were found in the QC in the postembryonic root, suggesting that SHR proteins move from the stele to adjacent cells, including the QC (Nakajima et al. 2001). Furthermore, it was observed that SHR protein is required for *SCR* activity in determining QC fate. It indicates a non-cell-autonomous function of SHR, also in the embryo, promoting the expression of *SCR* in the presumptive QC to determine its fate and maintain the stem cell niche (Aida et al. 2004). Notably, the expression of *SHR* and *SCR* does not depend on the PLTs (Aida et al. 2004). Further genetic analysis suggests that PLTs and SHR/SCR independently specify QC and stem cell niche.

### Transcriptional regulation of radial patterning

Radial patterning begins with a periclinal division that generates the protoderm, the outermost layer visible at the 16-celled embryo. TFs of the HD-ZIP class IV family, i.e., ARABIDOPSIS THALIANA MERISTEM LAYER1 (AtML1) and PROTODERMAL FACTOR2 (PDF2), are thought to control this cell differentiation. The process is marked by their expression as early as in the two-celled embryo, then later confined to the outermost layer in the subsequent developmental stages (Abe et al. 2003; Lu et al. 1996; Takada and Jürgens 2007; Iida et al. 2019). Whereas the single mutants have no apparent phenotypes, the double mutant (*atml1-1 pdf2-1*) embryos display defects in cell layer differentiation at the embryonic apex and a lack of epidermis in the leaves of their germinated seedlings (Abe et al. 2003). Moreover, the embryo development arrests around the globular stage in mutant combination with a strong allele of *AtML1* (*atml1-3*) (Ogawa et al. 2015). It indicates that AtML1 and PDF2 act redundantly for protodermal cell differentiation during embryogenesis.

Vascular identity is specified in the 16-cell embryo (Smit et al. 2020). A multigenic regulatory network, including TF-encoding genes, controls vascular tissue specification (Table 1). Among them, ARF5/MP and its interacting partner GBF2, a G-class bZIP TF, regulate the expression of many vascular-specific genes to specify the vascular tissue identity. GBF2 modulates and/or stabilizes the ARF5 binding to its target promoters (Smit et al. 2020). Subsequently, vascular initial cells undergo periclinal divisions to increase the number of cell files. It has been shown that ARF5 is also required for vascular tissue establishment and maintenance (De Rybel et al. 2013). Its mutant embryos contain fewer cell files due to abnormal periclinal divisions. ARF5 performs its function through its direct target, *TMO5,* and its closest homolog, *TMO5-LIKE1* (*T5L1*). Also, the heterodimer composed of TMO5 and another bHLH TF, LONESOME HIGHWAY (LHW), promotes periclinal divisions and establishes the vascular tissue in the embryo. In addition, the TMO5/LHW dimer controls the growth and patterning in the vascular tissue by regulating the expression of *LONELY GUY 4* (*LOG4*), required for the final step of cytokinin biosynthesis (De Rybel et al. 2014). This TMO5/LHW-LOG4 module creates distinct cytokinin and auxin response domains for growth and patterning (xylem and cambium) of the vascular tissue during embryogenesis.

There has been significant research on ground tissue patterning and maintenance in the postembryonic tissues around the SHR network. However, the molecular mechanisms for ground tissue initiation in the early embryo are still ambiguous. It was observed that ARF5/MP initiates the first ground tissue cells and controls their earliest asymmetric division in the early embryo (Möller et al. 2017). This activity of ARF5 does not depend on the SHR network genes. Also, it was observed that the SHR network is not required for the initiation of embryonic ground tissue cells. However, ARF5 transcriptionally regulates the expression of *SHR* and *SCR* in the early embryo, which indicates an unresolved role of these well-known regulators of ground tissue patterning in the embryonic ground tissue cells initiation (Möller et al. 2017). All TFs involved in embryo morphogenesis and mentioned in this review are compiled in Table 1.

**Table 1:** TFs involved in embryo morphogenesis

TF family	Involved TF	Function	References
WRKY	WRKY2	Zygotic polarity	Ueda et al. (2011)
WOX	WOX2	Controls apical fate; development of SAM	Breuninger et al. (2008) Haecker et al. (2004), Zhang et al. (2017)
	WOX8/9	Zygotic polarity; controls basal cell lineage	Breuninger et al. (2008), Haecker et al. (2004), Ueda et al. (2011)
	WOX1/3/5	Together with WOX2 regulate apical patterning and establishment of SAM	Breuninger et al. (2008), Haecker et al. (2004), Zhang et al. (2017)
HD-ZIP III	ATHB8, CNA, PHB, PHV, REV	SAM initiation and establishment of bilateral symmetry	Emery et al. (2003), Prigge et al. (2005), Smith and Long (2010)
	PHB, PHV, REV	Adaxial patterning	Emery et al. (2003), McConnell et al. (2001)
HD-ZIP II	HAT3, ATHB2, ATHB4	Establishment of bilateral symmetry	Turchi et al. (2013)
GARP/KAN	KAN1, 2, 4	Abaxial patterning	Eshed et al. (2004), Izhaki and Bowman (2007), Kerstetter et al. (2001)
AP2/ERF	DRN/DRNL	Interact with HD-ZIP III TFs and regulate cell patterning and cotyledon development	Chandler et al. (2007), Cole et al. (2009)
NAC	CUC1, 2, 3	Cotyledon boundary formation; act synergistically with STM to control SAM formation	Aida et al. (1999), Takada et al. (2001), Vroemen et al. (2003)
KNOX	STM	SAM formation	Aida et al. (1999), Takada et al. (2001), Vroemen et al. (2003)
B3	ARF5	Embryonic root formation; vascular tissue establishment and maintenance; ground tissue initiation	Berleth and Jurgens (1993), Crawford et al. (2015), De Rybel et al. (2013), De Rybel et al. (2014), Hamann et al. (1999), Hamann et al. (2002)
bZIP	GBF2	Cooperate with ARF5 during vascular tissue specification	Smit et al. (2020)
bHLH	TMO5, TMO7	Act downstream of ARF5 and control hypophysis specification	Schlereth et al. (2010)
	TMO5/T5L1, LHW	Vascular tissue establishment and maintenance	De Rybel et al. (2014)
AP2	PLT1, PLT2	Act downstream of ARF5 and ARF7, and regulate asymmetric division of hypophysis (QC specification)	Aida et al. (2004)
Zinc-finger	NTT, WIP4/5	Act downstream of ARF5 for QC specification	Crawford et al. (2015)
GRAS	SHR, SCR	QC identity and stem cells maintenance	Aida et al. (2004), Helariutta et al. (2000), Nakajima et al. (2001), Wysocka-Diller et al. (2000)
HD-ZIP IV	AtML1, PDF2	Protoderm differentiation	Abe et al. (2003), Iida et al. (2019), Lu et al. (1996), Ogawa et al. (2015)
YABBY	FIL, YAB2, YAB3	Abaxial patterning	Siegfried et al. (1999)

## Transcriptional regulation of seed maturation

**Fig. 3:**
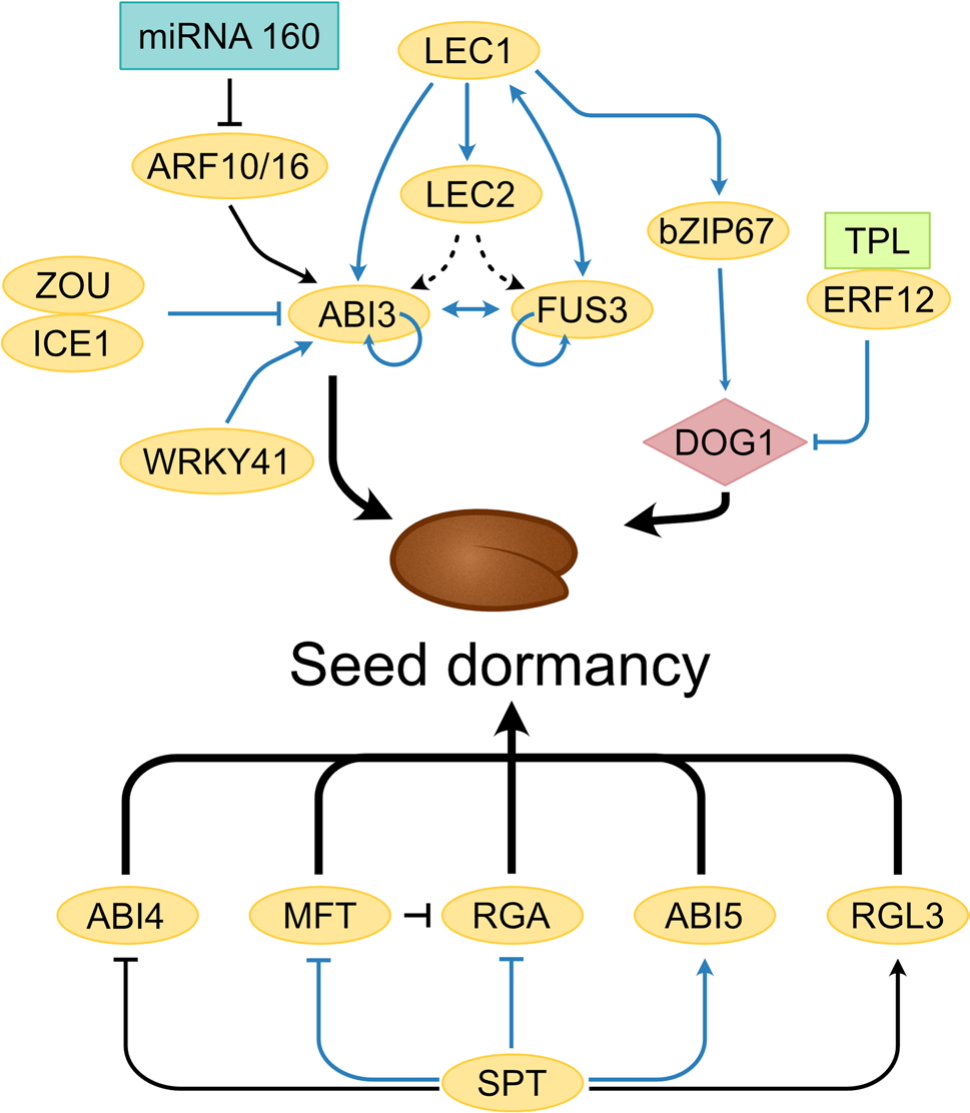
Regulatory network involving TFs in the regulation of seed dormancy. The regulation of seed dormancy by different transcription factors is shown. Arrows and T-shaped lines represent activation and repression, respectively. Direct targets are shown by blue lines. Dotted arrows indicate indirect regulation by To et al. (2006). SPT targets are from Vaistij et al. (2013). Thick arrows denote the promotion of seed dormancy

Seed maturation is a crucial phase of seed development as it ensures reproductive success by accumulating nourishment for the future seedling, helping to withstand desiccation, and promoting seed dispersal. In crop plants, including cereals and legumes, this phase is crucial for nutritional and economic purposes (Verma and Bhatia 2019; Gacek et al. 2018; Mathew et al. 2020). Maturation begins with the increase in the numbers of chloroplasts and chlorophyll accumulation (greening of the embryo), followed by the synthesis and accumulation of seed storage reserves (O'Neill et al. 2019; Baud et al. 2008). In oilseeds, like Arabidopsis, embryo greening appears necessary for photosynthesis and eventually accumulation of storage lipids (Mansfield and Briarty 1991). In Arabidopsis seeds, lipids as triacylglycerols (TAGs) and seed storage proteins (12S globulins and 2S albumins) are the major constituents of seed storage reserves (Baud et al. 2002, 2008). During late maturation, the embryo begins to lose water and chlorophyll (degreening) and simultaneously commences acquiring desiccation tolerance and dormancy (Leprince et al. 2017; Baud et al. 2008; Delmas et al. 2013). In Arabidopsis, the entire process of seed maturation has been shown under the tight control of several TFs that cooperate and act in a combinatorial manner (Fig. 3; Table 2; Boulard et al. 2017; Lepiniec et al. 2018; Jo et al. 2019).

**Table 2:** TFs involved in seed maturation

TF family	Involved TF	Function	References
B3	ABI3, FUS3, LEC2	Interact with each other; positively regulate the accumulation of seed storage reserves; embryonic fate; embryo maturation; seed dormancy; acquisition of desiccation tolerance	Braybrook et al. (2006), Braybrook and Harada (2008), Kagaya et al. (2005), Lepiniec et al. (2018), Nambara et al. (1995), To et al. (2006)
	HSI2/VAL1, HSL1/VAL2, VAL3	Repression of the LAFL network after germination	Chen et al. (2018), Suzuki et al. (2007), Tsukagoshi et al. (2007), Veerappan et al. (2012)
	ARF10, ARF16	Activate *ABI3* expression; seed dormancy	Liu et al. (2013), Tian et al. (2020)
NF-YB	LEC1	Embryonic fate; regulates storage protein and lipid accumulation; act as a molecular signal between endosperm and embryo; embryo maturation; desiccation tolerance	Jo et al. (2019), Lotan et al. (1998), Lepiniec et al. (2018), Song et al. (2021), Stone et al. (2008, 2001)
NF-YC	NF-YC2	FA biosynthesis	Mendes et al. (2013), Yamamoto et al. (2009)
bZIP	bZIP10, bZIP25, bZIP53	Cooperate with ABI3 in the regulation of SSP (At2S) gene expression	Alonso et al. (2009) Lara et al. (2003)
bZIP	bZIP67	Form trimeric complex with LEC1 and L1L, and activates expression of *CRUCIFERIN* and *FAD3*; acts downstream of LEC1 to establish seed dormancy	Bryant and Hughes (2019), Mendes et al. (2013), Yamamoto et al. (2009)
AGAMOUS-like	AGL15	Positive regulator of LEC2, ABI3 and FUS3 (seed maturation)	Chen et al. (2018)
	AGL67	Desiccation tolerance	González-Morales et al. (2016)
AP2	WRI1	Regulates genes involved in FA biosynthesis; direct target of LAFL	Kong et al. (2019), Maeo et al. (2009)
	ABI4	Seed dormancy; ABA signalling pathway	Shu et al. (2013)
R2R3 MYB	MYB89	Repression of *WRI1* and other essential genes involved in FA biosynthesis	Li et al. (2017)
TCP	TCP4	Negatively affects WRI1-mediated FA biosynthesis	Kong et al. (2020)
bHLH	TT8	Directly affects expression of *LEC1*, *LEC2* and *FUS3*; down-regulates the expression of genes involved in FA biosynthesis	Chen et al. (2014)
	MYC2, MYC3, MYC4	Control accumulation of SSPs and their relative proportion	Gao et al. (2016)
	SPT, ICE, ZOU	Seed dormancy	Vaistij et al. (2013) MacGregor and Zhang (2019)
E2F	E2FA, E2FB	Restrict premature accumulation of SSPs during early embryo development	Leviczky et al. (2019)
AP2/ERF	ERF12	Negatively affects seed dormancy	Li et al. (2019)
PLATZ	PLATZ1, PLATZ2	Desiccation tolerance	González-Morales et al. (2016)
WRKY	WRK41	Seed dormancy	Ding et al. (2014)

### LAFL transcription factors: the master regulators of maturation

To date, several TFs associated with the maturation phase have been identified and characterized in Arabidopsis. Among them, LAFL [LEAFY COTYLEDON1 (LEC1), ABSCISIC ACID INSENSITIVE3 (ABI3), FUSCA3 (FUS3), and LEC2] TFs have been demonstrated to play central roles in most aspects of seed maturation, including accumulation of seed storage reserves, ability to withstand desiccation, and establishment of dormancy (Boulard et al. 2017; Lepiniec et al. 2018; Jo et al. 2019). They are required for the onset of embryo maturation as they, at least partially, regulate chloroplast development and subsequent embryo greening, which is the first visible sign of embryo maturation (O'Neill et al. 2019). ABI3, FUS3, and LEC2 (hereafter referred to as B3-AFL) are the members of the B3 domain-containing TFs, whereas *LEC1* encodes the member of the NF-YB protein family (NF-YB9, HAP3 subunit of the CCAAT box-binding factors) (Braybrook and Harada 2008; Santos-Mendoza et al. 2008).

Mutations in *LAFL* genes revealed their partially overlapping functions during maturation (Meinke et al. 1994; West et al. 1994; Lotan et al. 1998; Nambara et al. 1995; Keith et al. 1994; Kagaya et al. 2005). However, they display some functional specificities. For instance, ABI3 controls chlorophyll degradation to mediate embryo degreening by regulating two functionally redundant genes, *STAY-GREEN1 (SGR1)* and *SGR2* (Delmas et al. 2013). Similarly, LEC1 and LEC2 have a distinct role in initiating and maintaining embryonic fate. Their ectopic expression is sufficient to induce somatic embryogenesis in vegetative tissues. And their loss-of-function mutants exhibit trichomes and anthocyanin accumulation on cotyledon’s surface (Lotan et al. 1998; Stone et al. 2008, 2001). Recently, it has been observed that the expression of *LEC1* in the embryo is not sufficient to initiate the maturation process. The endosperm-produced LEC1 protein, trafficked to the embryo through the suspensor, performs this function (Song et al. 2021). Moreover, detailed genetic analyses using complementation approach and mutant combinations of *LAFL* genes revealed the existence of a regulatory cascade with synergistic and/or redundant functions in parallel pathways (Bäumlein et al. 1994; Keith et al. 1994; Meinke et al. 1994; Parcy et al. 1997; To et al. 2006; Roscoe et al. 2015). Cross-regulation and feedback control of LAFL TFs have been observed. For instance, ABI3 and FUS3 regulate each other’s expression and can autoactivate themselves. Furthermore, their expression is positively regulated by LEC1 and LEC2 (To et al. 2006). One well-documented function of the LAFL network is to control protein and lipid accumulation during maturation (Roscoe et al. 2015; Baud et al. 2016). However, discrepancies in the level of control imposed by each LAFL on synthesis and accumulation of fatty acids and storage proteins have been observed (Baud et al. 2016; Roscoe et al. 2015; Kroj et al. 2003). For example, ABI3 exerts greater control over seed protein content, whereas FUS3 affects the lipid content to a greater extent (Roscoe et al. 2015). In another report, Baud et al. (2016) observed the strongest activation of an oleosin promoter fragment by ABI3 and the weakest by FUS3. Although LEC1 cannot activate the oleosin promoter alone, it enhances the synergistic activity of LEC2 and ABI3 on oleosin promoter expression. Oleosins are the structural proteins found on the surfaces of oil bodies of mature seeds (Frandsen et al. 2001).

Considerable efforts have been made to understand how *LAFL* genes control seed maturation. B3-AFL directly regulate maturation genes by binding to the RY element (CATGCA) and its variants found in many seed-specific promoters (Braybrook et al. 2006; Reidt et al. 2000; Kroj et al. 2003; Mönke et al. 2004; Baud et al. 2016; Sasnauskas et al. 2018). However, the binding specificity of the corresponding TFs depends on the flanking nucleotides of the core element (5′-CATG-3′) (Braybrook et al. 2006; Baud et al. 2016). Moreover, other motifs adjacent to the RY element are required to modulate this binding. In contrast, LEC1 does not exhibit specific DNA-binding activity. It interacts with NF-YA and NF-YC subunits to form the NF-Y complex, recognizing the CCAAT motif to regulate the transcription of its target genes (Dolfini et al. 2012; Baud et al. 2016). Nevertheless, an in vitro interaction of LEC1 with the *OLEOSIN1* (*OLE1*) promoter has been observed (Baud et al. 2016).

Moreover, these TFs act in concert with other TFs in the regulation of maturation-specific genes. For instance, ABI3 cooperates with the members of bZIP TFs such as bZIP10, 25, and bZIP53 in regulating SSP (At2S) gene expression (Alonso et al. 2009; Lara et al. 2003). Although ABI3 does not interact with bZIP53, it forms a ternary complex with the heterodimers of bZIP53 and bZIP10 or 25 to enhance the maturation gene expression. Likewise, LEC1 and its paralogs LEC1-like (L1L) interact with an NF-YC subunit and bZIP67. This trimeric complex (LEC1/L1L-NFYC2-bZIP67) binds to ABRE/G-box elements through bZIP67 to activate the expression of genes involved in storage, such as CRUCIFERIN and FATTY ACID DESATURASE3 (FAD3), an enzyme involved in fatty acid biosynthesis (Mendes et al. 2013; Yamamoto et al. 2009).

Direct and indirect targets of LAFL TFs have been identified using genome-wide approaches, including microarray-based profiling and chromatin immunoprecipitation (ChIP) assay (Wang and Perry 2013; Mönke et al. 2012; Tian et al. 2020; Pelletier et al. 2017; Braybrook et al. 2006). Several maturation-specific genes were identified as the targets of LAFL. Among them, the genes encoding albumins and oleosins appear to be regulated by all four TFs (Braybrook et al. 2006; Tian et al. 2020; Wang and Perry 2013). As mentioned above, LAFL regulate each other *in planta*. For instance, genomic regions of ABI3, LEC1, and FUS3 are directly bound by FUS3 (Wang and Perry 2013). ABI3, LEC2, and FUS3 have been identified as the direct targets of the LEC1/NF-Y complex (Pelletier et al. 2017). These findings further corroborate the genetic interactions that exist between LAFL (To et al. 2006). Moreover, ChIP tilling array highlighted the abundant presence of RY elements and G-boxes (CACGTG) in the ABI3- and FUS3-bound regions. This suggests that G-box elements modulate the known combinatorial activity of ABI3 and bZIP TFs on RY elements. However, the interaction of FUS3 with bZIP TFs in the regulation of seed storage proteins (SSPs) is not yet identified. Likewise, apart from the LEC1-binding CCAAT motif, other elements such as RY, ABRE-like, and G-box-like are overrepresented in LEC1-bound regions (Pelletier et al. 2017). Together, these findings imply that the combinatorial function of LAFL with other TFs is mediated by *cis*-regulatory modules containing the clustered binding sites of different TFs and by physical interactions that are known to occur between them.

The strict control of *LAFL* expression is crucial during the transition from seed maturation to seed germination and seedling growth. HIGH-LEVEL EXPRESSION OF SUGAR INDUCIBLE GENE2 (HSI2)/VIVIPAROUS1 ABI3-LIKE1 (VAL1), HSI2-LIKE1 (HSL1)/VAL2, and VAL3 mediate repression of the LAFL network, thereby restraining seed maturation program after germination (Tsukagoshi et al. 2007; Suzuki et al. 2007; Veerappan et al. 2012). Recently, Chen et al. (2018) reported that HSI2/VAL1 indirectly controls *LAFL* expression through down-regulation of *AGAMOUS-Like 15* (*AGL15*) during germination and seedling growth. Moreover, *AGL15* is also regulated by LAFL TFs. For example, *AGL15* is the direct target of LEC1 and FUS3, and its expression is induced by LEC2 (Braybrook et al. 2006).

### Other transcription factors regulating synthesis and accumulation of seed storage reserves

The AP2 domain-containing TF, WRINKELED1 (WRI1), is a crucial regulator of triacylglyceride (TAG) biosynthesis (Kong et al. 2019). It directly regulates genes involved in fatty acid (FAs) biosynthesis by binding to an AW-box present in their 5` upstream region (UTR) (Maeo et al. 2009). Genetic and molecular analysis has revealed that WRI1 acts downstream to LEC1 and LEC2 (Baud et al. 2007; Mu et al. 2008). Previously, FUS3 has been shown to affect the expression of fatty acid biosynthetic genes, probably by activating *WRI1* expression (Yamamoto et al. 2010). In recent reports, *WRI1* has appeared as the direct target of LEC1 and FUS3 (Wang and Perry 2013; Pelletier et al. 2017). Also, ABI3 directly induces *WRI1* and other fatty acid biosynthesis-related genes such as *FAD3* and *SSI2* (Tian et al. 2020). Another TF, MYB89, a member of the R2R3 MYB TF family, negatively regulates oil accumulation by directly repressing *WRI1* and other essential genes involved in oil synthesis and accumulation during maturation (Li et al. 2017). Three members of the TEOSINTE BRANCHED1/CYCLOIDEA/PROLIFERATING CELL FACTOR (TCP) TF family, i.e., TCP4, TCP10, and TCP24, have been identified as the interacting proteins of WRI1 (Kong et al. 2020). However, only interaction between TCP4 and WRI1 negatively affects WRl1-mediated oil biosynthesis in the seed.

TRANSPARENT TESTA GLABRA1 (TTG1), a WD40 repeat protein, suppresses the accumulation of seed storage reserves by negatively affecting the expression of genes encoding SSPs and enzymes involved in FA biosynthesis (Chen et al. 2015). It also indirectly suppresses the expression of genes that encode the major regulators of maturation and enzymes for the synthesis and modification of FAs. Moreover, TTG1 acts downstream to FUS3, which negatively regulates *TTG1* expression via direct binding. Similarly, TRANSPARENT TESTA8 (TT8), a bHLH TF, inhibits FA content by down-regulating the expression of genes involved in FA biosynthesis and directly affecting the expression of *LEC1*, *LEC2,* and *FUS3* (Chen et al. 2014). Likewise, three bHLH TFs, MYC2, MYC3, and MYC4, were found to redundantly control SSPs’ accumulation and their relative proportion in the seed. The loss-of-function triple mutants (*myc2 myc3 myc4*) accumulate fewer 2S albumins but more cruciferins (Gao et al. 2016). Recently, the E2F TFs, E2FA and E2FB, have been identified to act as repressors on the cell cycle genes and the maturation genes such as *LEC2* and *WRI1* during the transition from proliferation to maturation (Leviczky et al. 2019). They also restrict the premature accumulation of storage proteins during early embryo development.

## Transcriptional regulation of acquisition of desiccation tolerance and onset of dormancy

To survive extreme water loss, seeds need to develop desiccation tolerance (DT) during the mid-maturation phase. Like other seed maturation events, LAFL TFs are essential for seed DT. However, genetic evidence suggests that several other TFs act downstream of the LAFL network to control this process. González-Morales et al. (2016) identified genes that act downstream to the LAFL network to acquire DT using an integrated approach combining genetics, genomics, and metabolomics. The genes encoding three TFs, i.e., the plant AT-rich sequence and zinc-binding protein 1 (PLATZ1), PLATZ2, and AGL67, were shown to play a critical role in seed desiccation tolerance. Moreover, these TFs have been identified as the direct targets of ABI3 (Tian et al. 2020). In addition, many genes encoding Late Embryogenesis-Abundant (LEA) proteins that play a protective role during the acquisition of desiccation tolerance are directly induced by ABI3 (Tian et al. 2020; Kijak and Ratajczak 2020).

Primary seed dormancy (PD) is defined as the inability of a freshly matured seed to complete germination even under optimal conditions (Carrillo-Barral et al. 2020; Née et al. 2017). Seeds will germinate only after the release of dormancy by a period of dry storage at room temperature (after-ripening) or moist cold treatment (stratification) (Née et al. 2017). Some non-dormant seeds may experience a state of secondary dormancy upon exposure to unfavourable conditions such as very high or low temperatures or osmotic stress (Buijs 2020; Finkelstein et al. 2008). The network involving TFs in the establishment and control of seed dormancy is shown in Fig. 3.

The plant hormone abscisic acid (ABA) plays a central role in inducing and maintaining seed dormancy (Ali et al. 2021). Several mutants in which ABA signalling is attenuated display a reduced seed dormancy. Three TFs, including ABI3, ABI4, and ABI5, are the key downstream components of the seed-specific ABA response pathway. However, a loss-of-function mutation in *ABI3* and *ABI4*, but not in *ABI5,* reduces seed dormancy (Finkelstein 1994; Shu et al. 2013). A WRKY domain-containing TF, WRKY41, has been found to regulate PD by directly regulating *ABI3* expression in a maturing seed. However, its activity on *ABI3* appears to be independent of ABA signalling (Ding et al. 2014). ABI4, a member of the AP2 domain family, positively regulates PD by maintaining the balance between ABA and gibberellic acid (GA) biogenesis (Shu et al. 2013). Moreover, auxin also controls seed dormancy via activation of *ABI3* expression by two auxin-responsive TFs, ARF10 and ARF16 (Liu et al. 2013). MicroRNA160 post-transcriptionally regulates these two TFs. A feedback loop has been observed where ABI3 represses the expression of *miR160*, leading to increased transcripts of *ARF10* and *ARF16* that upregulate ABI3, resulting in seed dormancy (Liu et al. 2013; Tian et al. 2020). SPATULA (SPT), a bHLH TF, differentially regulates the establishment of seed dormancy in Arabidopsis ecotypes as Landsberg *erecta* (L*er*) seeds become highly dormant due to lack of *SPT*, whereas Columbia (Col-0) seeds are less dormant. SPT represses the expression of a gene encoding MOTHER-OF-FT-AND-TFL1 (MFT) that promotes seed dormancy. It interferes with ABA and GA signalling by repressing *ABI4* and *REPRESSOR-OF-GA* (*RGA*) expression, respectively. In contrast, it induces the expression of *ABI5* and *RGA-Like3 (RGL3)* to control primary seed dormancy (Vaistij et al. 2013). Two bHLH TFs, ZHOUPI (ZOU) and INDUCER OF CBF EXPRESSION1 (ICE1) are preferentially expressed in the endosperm and play a role in determining the depth of PD (MacGregor and Zhang 2019). Lack of ICE1 and ZOU activity increases seed dormancy that is accompanied by increased ABA levels. Moreover, ICE1 directly represses the expression of *ABI3*, thereby modulating the LAFL network to regulate seed dormancy.

The gene that encodes DELAY OF GERMINATION (DOG1) is essential for seed dormancy in Arabidopsis (Nonogaki 2019; Carrillo-Barral et al. 2020). It acts in parallel but independently to the ABA signalling pathway. The amount of active DOG1 protein determines the after-ripening period of freshly harvested seeds. Thus, it may serve as a timer for seed dormancy release. It was found that the *dog1-1* mutation can enhance the weak effects of the *abi3-1* allele, thus revealing the genetic interaction between ABI3 and DOG1 (Dekkers et al. 2016). This raises the possibility of ABI3 being at the point of convergence for both pathways. Another TF, bZIP67*,* acts downstream to LEC1 and regulates the expression of *DOG1* to establish primary seed dormancy (Bryant and Hughes 2019). ETHYLENE RESPONSE FACTOR12 (ERF12), a member of the AP2/ERF TF family, acts downstream of ETHYLENE RESPONSE1 (ETR1)/ REDUCED DORMANCY3 (RDO3) in the ethylene response pathway and negatively regulates seed dormancy by inhibiting the expression of *DOG1* (Li et al. 2019). It physically interacts with TOPLESS (TPL), and this complex binds to the DEHYDRATION-RESPONSIVE ELEMENT (DRE)/C-repeat (CRT) motif (5′-RCCGAC-3′) present on the promoter of *DOG1*.

## Methodologies used to identify TFs involved in embryo development

**Fig. 4:**
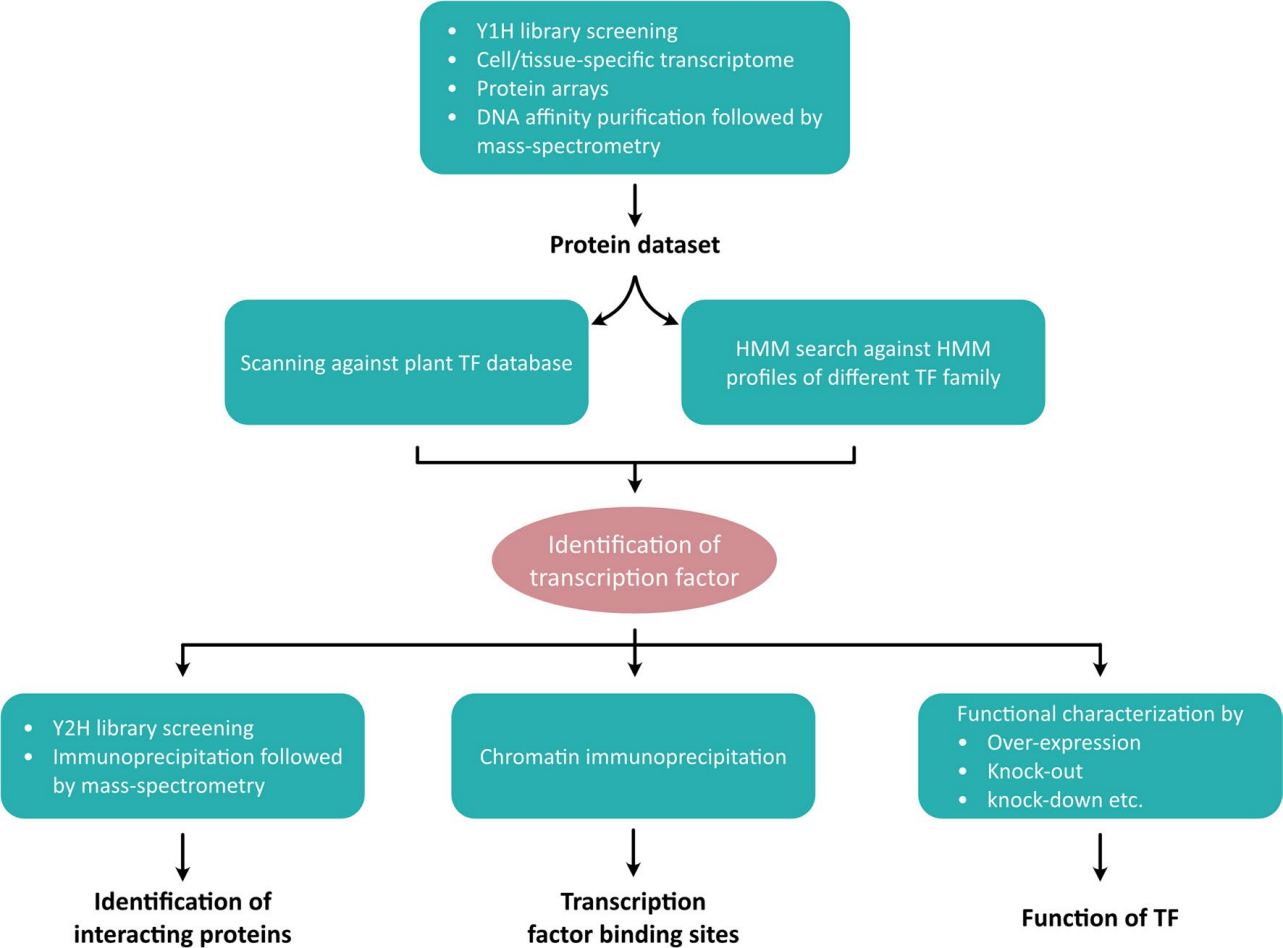
Genome-wide approaches for the identification of TFs and their molecular and functional characterization

Known and new TFs from different (plant and animal) tissues and developmental stages are often identified by methods such as yeast one-hybrid library screening (Reece-Hoyes and Marian Walhout 2012), transcriptome analysis (Andrilenas et al. 2015; Kodama et al. 2018), DNA affinity purification followed by Mass Spectrometry (Tacheny et al. 2013) and protein arrays (Hu et al. 2009; Fig. 4). The newly identified DNA-binding proteins are scanned against known plant TF databases such as the “Plant TF database” to annotate and assign them into a TF family (Jin et al. 2017). The classification of TFs under a specific family depends on the presence of DNA binding domain (DBD) in their protein sequence that shows sequence homology to the previously characterized DBDs such as B3, NAC, C2H2, AP2, etc. These DBDs are catalogued in different databases such as InterPro (Blum et al. 2021), Pfam (Mistry et al. 2021), SMART (Letunic et al. 2021) as multiple sequence alignments, and hidden Markov models (HMM). Alternatively, these HMM profiles can be used to scan the protein dataset for the presence of a particular DBD, thereby classifying the proteins into a corresponding TF family. Indeed, transcriptome data provide valuable information on the gene expression pattern. Potential candidate TFs having differential expression can be identified by comparing transcriptomes of different tissues and/or different developmental stages. For Arabidopsis, such expression profiles can be found in the gene expression tool, ePlant (http://bar.utoronto.ca/eplant/). In addition, putative TFs regulating different sets of genes can be identified by performing co-expression analysis (Zogopoulos et al. 2021). These methods have successfully been utilized in the past decade to identify TFs involved in seed and/or embryo development in different plant species (Wu et al. 2020; Gu et al. 2020; Pradhan et al. 2014; Yi and Gu 2019). The identified TFs can be molecularly characterised using genomic approaches to identify binding promoters (e.g., ChIP assay) and to identify interacting proteins (e.g., yeast two-hybrid library screening, Mass spectrometry).

Among the methods mentioned above, transcriptomics has gained widespread interest in identifying gene regulatory networks, including TFs. However, most transcriptomics were performed utilizing whole or parts of embryos. It limits the understanding of transcriptome changes at the cellular level during early embryogenesis. To identify TFs for cell fate specification, the utilization of cell-type-specific transcriptome would be a better approach. Various methods such as laser capture microdissection (LCM), fluorescence-activated cell sorting (FACS), translating ribosome affinity purification (TRAP), and isolation of nuclei tagged in specific cell types (INTACT) are available to isolate specific cell types for transcriptome profiling (reviewed in Palovaara et al. 2013).

Although significant transcriptome profiling has been carried out to study seed/embryo development, only a few have focused exclusively on TFs required for the development of different stages and/or tissues of seeds. For example, Le et al. (2010) identified 48 seed-specific TFs using Affymetrix Gene Chips. These TF genes exhibit stage-specific expression, suggesting their active involvement in that particular stage. Similarly, 57 candidate TFs have been identified using RNA-seq data of developing embryos (Hofmann et al. 2019). In addition to known TF genes, some novel TFs involved in embryo morphogenesis were identified, such as storekeeper protein-related transcripts, MYB62, and REGULATOR OF AXILLARY MERISTEMS2 (RAX2) (Hofmann et al. 2019). Likewise, distinct TFs involved in the apical and basal cell lineages specification have been identified using the RNA-seq data of the apical and basal domains of the proembryo (Zhou et al. 2020). Differential expression profiling of the identified TFs resulted in 73 and 39 apical and basal cell lineage-maintained TFs, respectively. Moreover, putative TFs that may regulate the other lineage-specific genes were identified by co-expression analysis and by analysing the promoter sequences of lineage-specific genes for the presence of TF binding motifs. Recently, using single-nucleus mRNA-sequencing, Kao et al. (2021) identified many candidate TFs involved in the development of different cell types of early embryos.

Besides, a yeast-one hybrid library screening identifies a repertoire of TFs that can bind to the DNA region of interest. However, utilization of this method for genome-wide identification of TFs associated with seed development is very limited in Arabidopsis. Recently, Smit et al. (2020) identified potential TFs that may contribute to vascular identity during embryogenesis using a large-scale enhanced yeast one-hybrid assay. Among the most robust candidate TFs, the G-class bZIP TF G-box-binding factor 2 (GBF2) was identified as an interacting protein of MP/ARF5 in modulating vascular gene expression.

The identified TFs can be molecularly characterised using genome-wide approaches to identify binding promoters (e.g., ChIP assay) and interacting proteins (e.g., yeast two-hybrid library screening, Mass spectrometry). Furthermore, reverse genetics methods are widely used to establish the function of identified TFs, such as generating mutants and over-expression lines of newly identified genes, followed by phenotype analysis. Non-targeted gene mutations are achieved by chemical mutagenesis, transgene and transposon insertions. These methods are generally used to knock out a gene where the gene is completely deactivated. For Arabidopsis, such mutants may be available to researchers through seed stock centers. In the last few years, type II CRISPR/Cas (CRISPR/Cas9) has emerged as a powerful genome-editing tool for targeted gene mutations and knocking out the function of a gene (Liu et al. 2017). A modified CRISPR/Cas9 system can also be used for transcriptional activation and repression in plants (Piatek et al. 2015). Furthermore, RNA interference (RNAi) is a potential method for gene knockdown where an exogenous or endogenous small double-stranded RNA (dsRNA) interferes with target gene expression (Hung and Slotkin 2021). In addition, over-expression lines are utilized to analyze the effects of gain-of-function of studied genes. However, over-expression of a gene using constitutive promoters may harm the plant, resulting in seed/embryo abortion, lack of germination, or reduced seed set. To overcome such issues, tissue-specific (restricted) promoters and/or inducible gene expression systems can be used (Borghi 2010). For example, to achieve early-embryo-specific expression, *WOX2* promoter has been used in Arabidopsis, whereas the promoters of seed storage proteins have been a major choice to study the maturation stage (Liao and Weijers 2018; Jeong et al. 2014).

## Conclusions and perspectives

It is evident that TFs are crucial members of regulatory networks involved in many biological processes, including seed development. Therefore, the mechanisms by which these TFs exert their function, including interactions with other proteins and target promoters, will provide strategic insights toward understanding this complex developmental process. To date, several TFs involved in seed development have been identified using forward and reverse genetic analysis. Nevertheless, their molecular and genetic interactors remain to be explored. To achieve this goal, it is essential to utilize global approaches such as affinity purification followed by mass spectrometry and ChIP assays, especially using seed tissues. For example, in recent years, using ChIP coupled with transcriptomics, the target genes of many TFs began to emerge to a greater extent. Such techniques would be helpful to uncover several new genes that would aid in understanding and manipulating the regulatory mechanisms involved in seed development.

Although many TFs involved in maturation have been identified and characterized at the molecular and genetic levels, knowledge on the transcriptional regulation of early embryogenesis is still minimal. For instance, most studies are biased towards the ARF5 TF for lower tier domain fate. Some other TFs most probably exist in this process that need to be detected. Also, hormones such as auxin and ABA are required for early embryogenesis and maturation. Identifying TFs involved in their biosynthesis and transport would open the door to devising new strategies to control these processes of seed development. Moreover, the activity of TFs is tightly controlled by several genetic and epigenetic factors, the knowledge of which is still fragmented. Systemic analysis of such factors will aid in expanding regulatory networks and filling the knowledge gaps for better comprehension of seed development. Furthermore, to deepen our knowledge, cutting-edge approaches such as cell-type-specific transcriptomics are available to identify cell and/or tissue-specific TFs in developing embryos. Overall, this review provides a repository of TFs as potential candidates that can be functionally studied in crop plants to improve seed quality and agronomic practices.

### *Author contribution statement*

SV reviewed the literature and wrote the manuscript. VPSA prepared the illustrations. HSR reviewed and edited the manuscript. All authors read and approved the manuscript.

## Data Availability

Data sharing does not apply to this article as no datasets were generated or analyzed during the current study.
